# Rare-earth doped BiFe_0.95_Mn_0.05_O_3_ nanoparticles for potential hyperthermia applications

**DOI:** 10.3389/fbioe.2022.965146

**Published:** 2022-10-18

**Authors:** Astita Dubey, Soma Salamon, Supun B. Attanayake, Syaidah Ibrahim, Joachim Landers, Marianela Escobar Castillo, Heiko Wende, Hari Srikanth, Vladimir V. Shvartsman, Doru C. Lupascu

**Affiliations:** ^1^ Institute for Materials Science and Center for Nanointegration Duisburg-Essen (CENIDE), University of Duisburg-Essen, Essen, Germany; ^2^ Faculty of Physics and Center for Nanointegration Duisburg-Essen (CENIDE), University of Duisburg-Essen, Duisburg, Germany; ^3^ Department of Physics, University of South Florida, Tampa, FL, United States

**Keywords:** bismuth ferrite, nanoparticles, rare-earth, hyperthermia, doping, multiferroics, piezoresponse, magnetic

## Abstract

Ionic engineering is exploited to substitute Bi cations in BiFe_0.95_Mn_0.05_O_3_ NPs (BFM) with rare-earth (RE) elements (Nd, Gd, and Dy). The sol-gel synthesized RE-NPs are tested for their magnetic hyperthermia potential. RE-dopants alter the morphology of BFM NPs from elliptical to rectangular to irregular hexagonal for Nd, Gd, and Dy doping, respectively. The RE-BFM NPs are ferroelectric and show larger piezoresponse than the pristine BFO NPs. There is an increase of the maximum magnetization at 300 K of BFM up to 550% by introducing Gd. In hyperthermia tests, 3 mg/ml dispersion of NPs in water and agar could increase the temperature of the dispersion up to ∼39°C under an applied AC magnetic field of 80 mT. Although Gd doping generates the highest increment in magnetization of BFM NPs, the Dy-BFM NPs show the best hyperthermia results. These findings show that RE-doped BFO NPs are promising for hyperthermia and other biomedical applications.

## Introduction

Magnetic nanoparticles (NPs) are known as potential candidates for hyperthermia cancer treatment, which is an efficient technique with minimal adverse effects on the patients. For treating cancer, hyperthermia exploits the heat released by magnetic nanoparticles when exposed to an alternating magnetic field to shock or kill cancer cells. Cancer cells can die if their temperature reaches the 40–44°C window. If not, hyperthermia will at least make the primary treatment processes, such as radiotherapy, more effective by making the cancer cells more sensitive to radiation and certain drugs. (Bañobre-López, Teijeiro and Rivas, 2013), (Nemati et al., 2017), (Das et al., 2021) Specific loss power (SLP) is a parameter that determines the heating efficiency of NPs, determined by calculating the power dissipated per unit mass of material. SLP depends on the magnetization, the magnetic anisotropy of the material, viscosity, the concentration of NPs in the dispersion, and the amplitude and frequency of the applied field. Another important factor is the width and overall shape of the magnetization hysteresis loops. In addition, NPs with a higher atomic number, i.e., higher X-ray absorption than living tissues, can be useful to increase the effect of radiation within cancer cells and reduce the amount of material for the same effect.

Bismuth based materials have been utilized in many biomedical applications like skin lesions, syphilis treatment (Salvador et al., 2012) and as CT contrast agents (Wei et al., 2016). Bismuth ferrite (BiFeO_3_; BFO) NPs are multifunctional materials due to their wide applicability in the fields of photocatalysis, photovoltaics, sensors, radiosensitizers, and radiotherapy. (Rajaee et al., 2019), (Dubey et al., 2021), (Chen et al., 2019) BFO is mainly known as a rare room-temperature single phase multiferroic, (Catalan and Scott, 2009; Kadomtseva et al., 2006), but its low toxicity, low cost and chemical stability in ambient conditions make it promising for biomedical applications. For example, bio-compatible BFO NPs have been studied as radio-thermotherapeutic agents to enhance the theranostic efficiency. (Feng et al., 2020), (Staedler et al., 2015) It is believed that the magnetic moment, large ferroelectric polarization, and ultimately the magnetoelectric coupling between them are beneficial for the biomedical applications. Therefore, much effort has been made to improve the ferroelectric and magnetic properties of BFO. (Gebhardt and Rappe, 2018; Yang et al., 2012). The increase of magnetic moments by reducing the particle size of BFO (Park et al., 2007; Castillo et al., 2013), has been one of the remarkable tasks which opens the door of utilization of BFO NPs in hyperthermia therapy.

Unfortunately, BFO NPs show smaller magnetization and relatively smaller coercive field in comparison with the iron oxides. The magnetic structure of bulk BFO is characterized by an incommensurate cycloidal modulation of the weak magnetic moment arising due to the canting of the antiferromagnetically ordered magnetic moments of the neighbouring Fe^3+^ ions. Therefore, the net magnetization of bulk BFO is cancelled. On the contrary, BFO NPs show non-zero magnetization, which arises from uncompensated spins at the surface and distortion of spin cycloid in the particles with diameter less than the period of the cycloid of 62 nm in the bulk. (I. Sosnowska et al., 1992). To further increase the magnetization of BFO NPs, the ionic substitution at Bi and Fe sites has been found to be the most effective and convenient way. (Silva et al., 2011), (Dubey et al., 2019) Usually, Fe is substituted by transition metals. (Salak et al., 2021). Among them, manganese is of particular importance due to its own magnetic moment and multiple oxidation states. Previously, we found that introduction of even 5 mol% Mn into Fe sites significantly increases the magnetization of BFO NPs due to the destruction of the cycloidal spin modulation. (Dubey et al., 2020), (Dubey et al., 2019). This makes Mn-doped BFO NPs better candidates for hyperthermia applications than the pure BFO NPs.

Into the Bi-site, usually alkaline earth and rare-earth (RE) metals are incorporated, (Khomchenko et al., 2009), (Gebhardt and Rappe, 2018), (Silva et al., 2011), (Arnold, 2015) where substitution for RE metals leads to the strong increase in magnetization. On the one hand, this is due to the own magnetic moment of rare-earth elements. On the other hand, the mismatch between the sizes of the Bi^3+^ and RE^3+^ cations leads to local stresses that affect the Fe-O-Fe bond angle and, consequently, the superexchange interaction and the magnetic state of NPs. For example, doping with Nd up to 20% into BFO NPs increases the maximum magnetization up to 0.8 Am^2^/kg at room temperature (RT) (Li et al., 2015). In another report, Nd doping up to 10% in bulk BFO, increases the maximum magnetization up to 0.12 Am^2^/kg at RT (Chang et al., 2017). 10–30% Gd doping results in a significant increase in magnetization with decreasing temperature. (Khomchenko et al., 2009). A magnetization of 2 Am^2^/kg was reported for 15% Gd doped BFO NPs at RT. (Guo et al., 2010). Similarly for 20% Dy doped BFO NPs, a magnetization of 0.7 Am^2^/kg was reported at RT (Pattanayak et al., 2014). Different types of dopants also influence the morphology and size of NPs. The magnetic anisotropy of the NPs depends upon their crystal orientation, and shape.

Previous reports on RE metal doped BFO NPs have focused only on magnetic properties at higher doping concentrations, whereas the role of different RE dopants and their application for hyperthermia tests have not been discussed in detail. In addition, probing the ferroelectric properties is also not a direct and easy task for NPs. Therefore, ferroelectric, and piezoelectric properties of RE-doped BFO NPs still lack systematic and overall investigation. This motivated us to research the magnetic and ferroelectric properties of RE-doped BFO NPs and their utilization in hyperthermia tests.

We present a comparative study among three different RE metals: Nd, Gd, and Dy as dopants (5 mol%), to study their influence on the magnetic and ferroelectric properties of BiFe_0.95_Mn_0.05_O_3_ NPs (BFM). In our previous study, we showed that 5 mol% Mn incorporation at the Fe site increases the magnetization of BFO NPs. (Dubey et al., 2020), (Dubey et al., 2019) The choice of the RE-dopants was dictated by the different configuration of their outer electronic shells: less than half-filled *f*-orbitals (Nd^3+^), half-filled *f*-orbitals (Gd^3+^), and more than half-filled *f*-orbitals (Dy^3+^). Consequently, these ions have significantly different magnetic moments and differently affect the magnetic properties of BFO NPs. The RE-doped NPs are used for hyperthermia tests, where Dy-BFM shows the best properties among all. We observe that Nd-BFM NPs show highest piezoresponse and so best ferroelectric properties among all.

## Experimental section

### Materials

Bismuth nitrate Bi(NO_3_)_3_·5H_2_O (≥98%), iron nitrate Fe(NO_3_)_3_·9H_2_O (≥98%), manganese acetate Mn(CH_3_COO)_2_·4H_2_O (≥99%), neodymium nitrate Nd(NO_3_)_3_·6H2O (≥99%), gadolinium nitrate Gd(NO_3_)_3_·6H_2_O (99.9%), dysprosium nitrate Dy(NO_3_)_3_·xH_2_O (99.9%), tartaric acid C_4_H_4_O_6_ (≥99%), and nitric acid HNO_3_ (65%) were purchased from Sigma Aldrich. RE and Mn co-doped BFO NPs (Bi_0.95_A_0.05_Fe_0.95_Mn_0.05_O_3_: A = Nd, Gd, Dy) were synthesized by a modified wet chemical ‘sol-gel route’ followed by a calcination step (500°C, 1 h) as described elsewhere. (Dubey et al., 2020). The sol-gel method is an easy synthesis route that requires low processing temperature and mild chemical conditions, producing materials of high purity with a high degree of reproducibility.

For the synthesis, 0.01 mol of Bi(NO_3_)_3_·5H_2_O and Fe(NO_3_)_3_·9H_2_O were dissolved in 25 ml of 2 N HNO_3_ with 0.02 mol of tartaric acid. A sol was formed after keeping it under continuous magnetic stirring at room temperature for at least 10 h. The yellowish sol was heated up to 100°C and kept at this temperature for 5 h. The xerogel was collected and ground in a mortar, followed by a calcination process at 500°C for 1 h with 3 K/min heating and cooling rates to obtain the NPs. For doped BFO NPs, we followed the same method with dopant precursors added to the mother liquor in the corresponding molar percentages.

We have used the following abbreviations for the NPs under study [BiFeO_3_: BFO, BiFe_0.95_Mn_0.05_O_3_: BFM, Bi_0.95_Nd_0.05_Fe_0.95_Mn_0.05_O_3_: Nd-BFM; Bi_0.95_Gd_0.05_Fe_0.95_Mn_0.05_O_3_: Gd-BFM; Bi_0.95_Dy_0.05_Fe_0.95_Mn_0.05_O_3_: Dy-BFM].

## Materials and methods

### X-ray diffraction (XRD)

The phase analysis and lattice parameters of all NPs were studied by powder XRD using a Panalytical Empyrean (Cu Kα radiation) diffractometer over a 2θ range of 10°–80° with a step size of 0.026°. For measurements, the powder was placed on a standard sample holder and measured in reflection mode (Bragg-Brentano geometry). The background parameters, zero shift and detector shifts, lattice parameters and atomic coordinates for the host and doping atoms, and profile parameters (Pseudo-Voight function including crystallite size, and strain parameters) were fitted simultaneously using the Rietveld refinement procedure realized in the High Score Plus software.

### X-ray photoelectron spectroscopy (XPS)

XPS measurements were performed on a VersaProbe II System by UlvacPhi. A monochromatized Ag-Kα source was used with a beam diameter of 100 µm. The NPs were dropcasted onto a conductive Si-wafer for the measurements. For data analysis a Shirley background was used. The precise way of fitting depends on the element. In the case of carbon, the Gaussian-Lorentzian curve is representative for one specific species (the same applies for oxygen and silicon).

### Transmission electron microscopy (TEM)

The size and crystallinity of the NPs are analyzed using high-resolution TEM on a JEOL JEM-2200FS microscope using a 200 kV acceleration voltage and a probe side aberration corrector. The atomic composition of the NPs was estimated by energy dispersive X-ray spectroscopy (EDXS) and high angle annular dark field imaging (HAADF) using an Analytical scanning electron microscope (SEM) device (EDXS, energy resolution < 132 eV for Mn Kα, detector area 10 mm^2^).

### Piezoresponse force microscopy (PFM)

To probe the ferroelectric properties of the NPs, PFM measurements were performed using a commercial scanning probe microscope MFP-3D (Asylum Research). The NPs were drop cast onto a conductive carbon tape for the PFM measurements. Pt/Cr coated cantilevers Multi 75E-G (Budget Sensors) with a spring constant of 3 N/m were used. PFM measurements were conducted at a probing voltage amplitude *U*_ac_ = 10 V and frequencies *f* = 400 kHz, and 700 kHz for the vertical PFM (VPFM) and lateral PFM (LPFM) modes, respectively.

### Magnetometry

Magnetic measurements were performed using the vibrating sample magnetometer (VSM) option of a Quantum Design PPMS DynaCool at 300 K and at 5 K up to a maximum magnetic field of 9 T. Foremost a 9 T *M*(*H*) sweep at 300 K was recorded, followed by the 300–900 K *M*(*T*) sweep at 0.1 T, and lastly another *M*(*H*) sweep at 300 K. All samples were measured at 5 K/min rate for *M-T* plots. The NPs were pressed into 3 mm discs prior to weighting and then glued onto a ceramic heater with the help of a cement (Zircar AL-CEM). The high temperature *M*(*T*) measurements were performed in high vacuum (below 10^−5^ mbar), while the sample space was repeatedly purged with He gas and pumped down after each sample change.

### Hyperthermia tests

The experiments were carried out using a 4.2 kW Ambrell Easyheat Li3542 system with a frequency of the AC magnetic field of 310 kHz. The NPs were dispersed into both water and agar media. Agar was used because it roughly has the same viscosity as living cells. 1 mg/ml and 3 mg/ml concentrations of NPs were used for the testing at two different AC fields of 60 mT and 80 mT.

## Results

**FIGURE 1:**
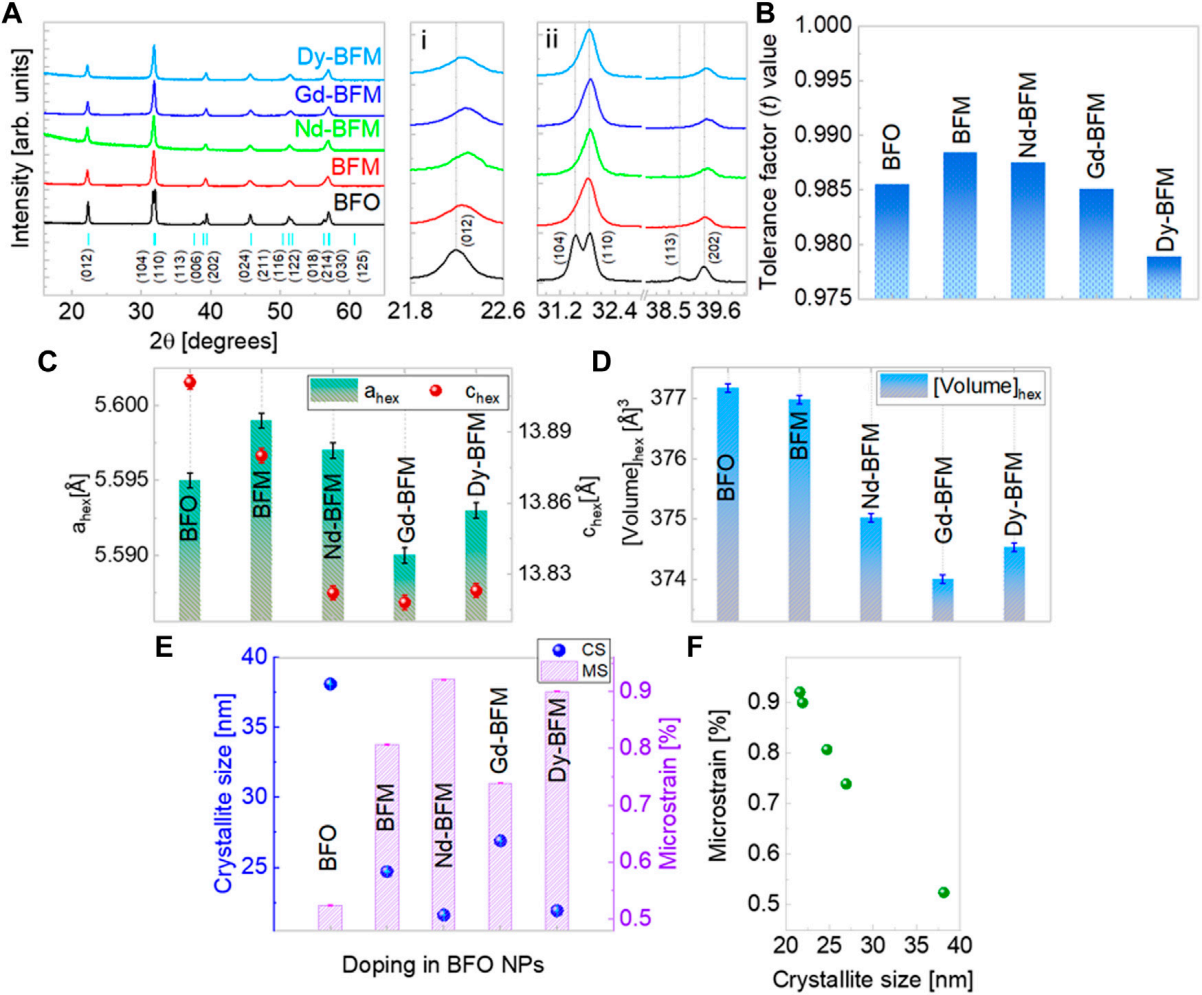
X-ray diffractograms of rare earth (Nd, Gd, Dy) doped BFM NPs (**A**, i, ii) along with peak indexing based on ICSD database. Tolerance factor trend in doped BFO NPs **(B)**, lattice parameters variation in hexagonal structure **(C)**, unit cell volume **(D)**, and crystallite size and microstrain values in doped BFO NPs **(E)**, Linear relation between microstrain and crystallite size of NPs **(F)**.

The XRD results shown in Figure 1 confirm the single perovskite phase of both undoped and doped NPs under the XRD detection limit. To probe the crystal structure of RE doped BFM NPs, Rietveld refinement was performed on the XRD diffractograms. As per the phase analysis, all NPs have a rhombohedral crystal structure (space group: *R*3*c*). A slight shift in the diffraction peak positions towards larger 2θ angle and the merging of some of the peaks (e.g., (104)/(110) doublet at 2θ ≈ 32° and (113)/(006)/(202) triplet at 2θ ≈ 39°) [Figure 1 i-ii] compared with the pure BFO NPs manifest an incorporation of the dopants into the crystal lattice. The distortion in the crystal structure can be predicted by calculating the Goldschmidt’s tolerance factor (*t*) of these perovskites, (V. Goldschmidt, T. Barth, G. Lunde, 1926) (Figure 1B) where we find that the *t* value of BFM NPs decreases from Nd to Dy doping into BFM NPs. The size trend of the RE ions is: **Bi** ^**3+**^ **(1.45 Å) > Nd** ^**3+**^ **(1.41 Å) > Gd** ^**3+**^ **(1.27 Å) > Dy** ^**3+**^ **(1.18 Å)** as for the 12-fold coordination of the A-site in the cubic perovskite structure. (Shannon, 1976). As the *t* value decreases, the FeO_6_ octahedron distorts respective to the octahedron in the ideal perovskite structure with *t* = 1 (Maughan et al., 2018).

The variation of the lattice parameters *a*_*hex*_ and *c*_*hex*_ reflects a distortion in the crystal structure of BFM NPs due to RE doping at Bi site [Figure 1C]. The unit cell volume decreases as per RE doping but not linearly as shown in Figure 1D. Gd-BFM has the least unit cell volume, due to decreased *a*_*hex*_ and *c*_*hex*_ values, whereas, Nd-BFM has maximum unit cell volume among the RE doped NPs. Doping of larger ions like Ba^2+^, reduces the crystallite size of BFM NPs (Dubey et al., 2020). We observe that there is reduction in the crystallite size of BFM NPs upon Nd and Dy doping, however the crystallite size increases upon Gd doping [Figure 1E]. In addition, Nd and Dy dopants induce more microstrain into BFM NPs than Gd [Figure 1F].

**FIGURE 2:**
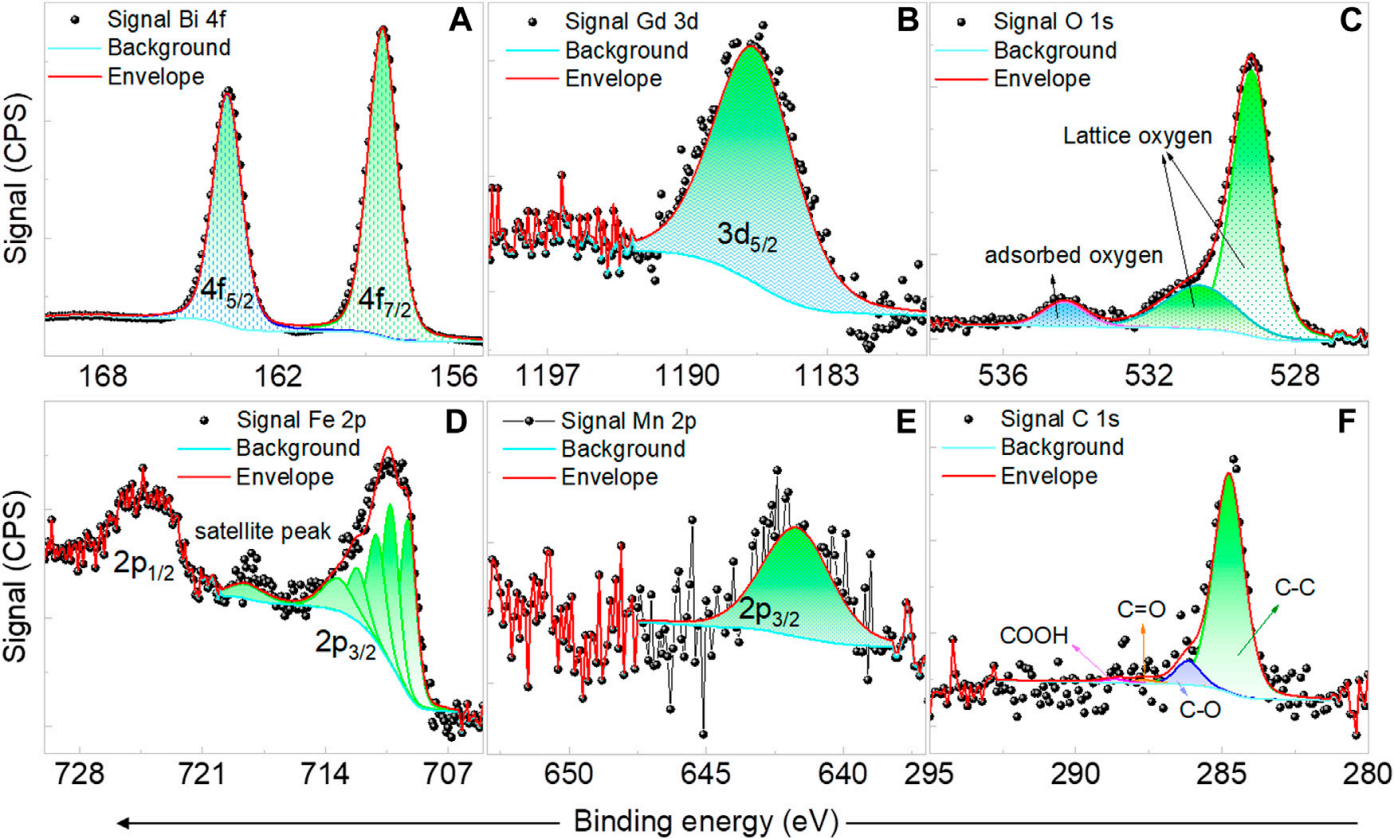
Representative XPS spectra of Bi 4f **(A)**, Gd 3d **(B)**, O 1s **(C)**, Fe 2p **(D)**, Mn 2p **(E)**, and C 1s **(F)** for Gd and Mn co-doped BFO NPs.

The further confirmation of dopants’ incorporation and their oxidation states are done using XPS. A representative XPS spectrum of Gd-BFM NPs is shown in Figure 2. From the 4f XPS spectra of Bi, its 3+ state is confirmed in all NPs [Figure 2A]. From the oxygen 1s spectra, the 2- state of oxygen is observed in all NPs [Figure 2C]. In 1s spectra of oxygen, the peaks at ∼529 eV and ∼530 eV correspond to lattice bounded oxygen. The peaks at energies higher than 531 eV correspond to adsorbed ^−^OH groups. We find that such peaks are more pronounced in RE-BFM than in BFM and BFO, which can be explained by lanthanide reactivity towards air and moisture (Barreca et al., 2007). The formed carbonates and bicarbonates observed in the carbon 1s XPS spectra of Gd-BFM also confirm the high reactivity of RE dopants. The Fe-3p spectra of Gd-BFM, manifest mostly 3+ state of Fe [Figure 2D]. From the Mn-2p spectra [Figure 2E], we observed Mn^4+^ as the main oxidation state whereas a slight amount of 3+ state cannot be excluded. From 3d spectra of Gd, its 3+ state is confirmed [Figure 2B]. Though this analysis is for the surface of NPs as XPS unveils largely the surface chemistry than bulk.

**FIGURE 3:**
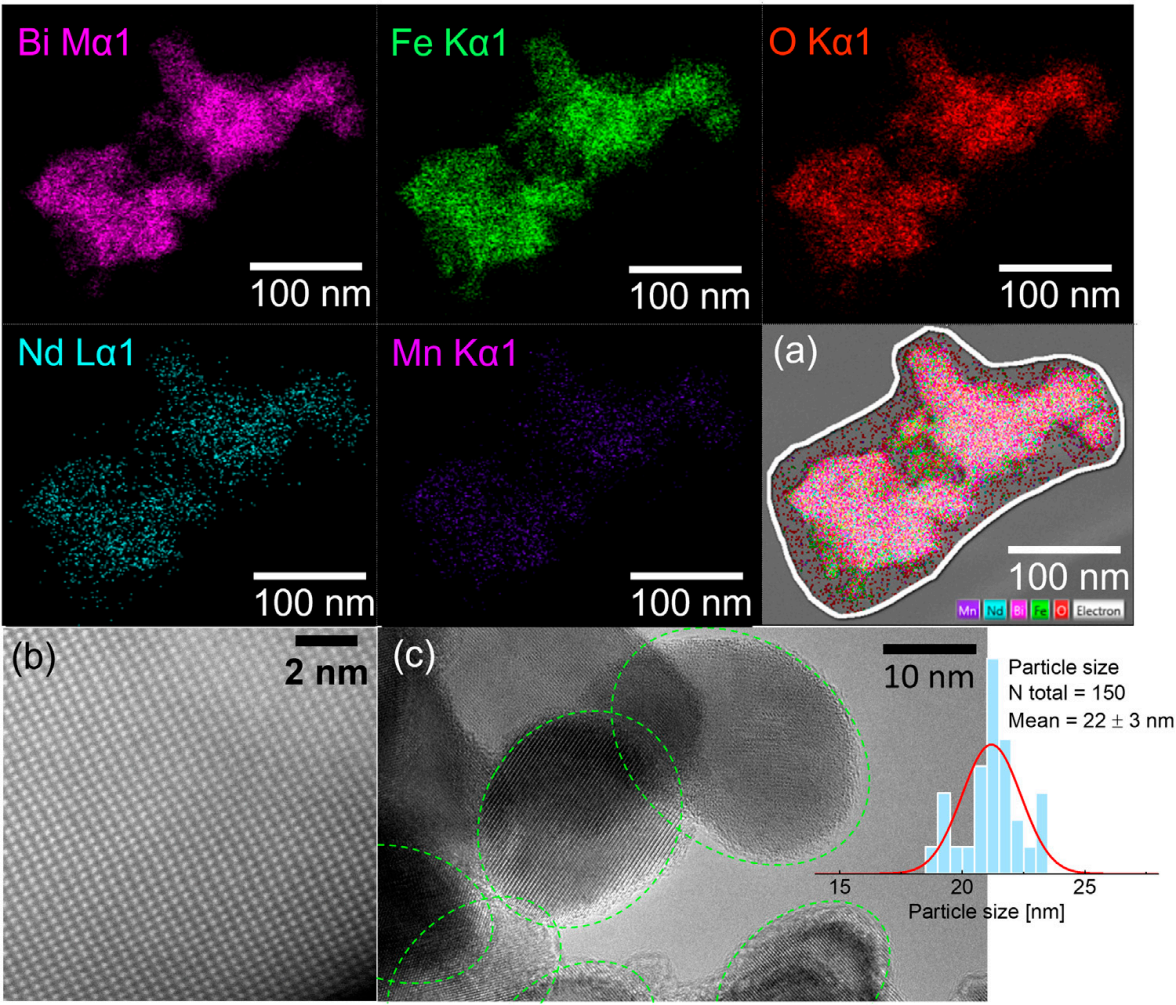
Representative HAADF images of the elemental distribution in Nd and Mn co-doped BFO NPs shown by different colors. The overlay of elements in a chosen area of agglomerated NPs **(A)**, STEM image of Bi-atom’s arrangement in the NPs, shows high crystallinity **(B)**, and HRTEM image along with particle size distribution histogram for Nd and Mn co-doped BFO NPs **(C)**.

The elemental analysis was additionally confirmed *via* EDXS, the results are tabulated in Supplementary Table S1. As can be seen the host and the doping elements are in the expected stochiometric ratio according to the doping amount of 5 mol% during synthesis. Even the dopants are found to be homogeneously distributed throughout the particles shown in HAADF images [Figure 3]. The elements are shown by different colours in the overlay image collected by the HAADF mode of TEM [Figure 3A].

The morphology and particle size of NPs were analyzed using TEM images. Representative TEM images of Nd-BFM NPs are shown in Figure 3. The Nd-BFM NPs are highly crystalline as it can be seen in Figure 3B, where bright spots correspond to Bi atoms which are well arranged in the 3D crystal lattice of BFO. Most of the Nd-BFM NPs have an elliptical shape, in contrast to the irregular pentagon-shaped Dy-BFM NPs and rectangular Gd-BFM NPs. The shape and average particle size of the NPs are compared and tabulated in Supplementary Table S2. The particle sizes of the RE-doped BFM NPs are smaller than those of BFM. The different morphology of the doped-NPs can lead to their different magnetic anisotropy.

As BFO and BFM NPs are multiferroic materials (Dubey et al., 2020), our purpose is not only to study their magnetic properties, but to also to determine what happens to their ferroelectric response upon doping.

### Ferroelectric properties

**FIGURE 4:**
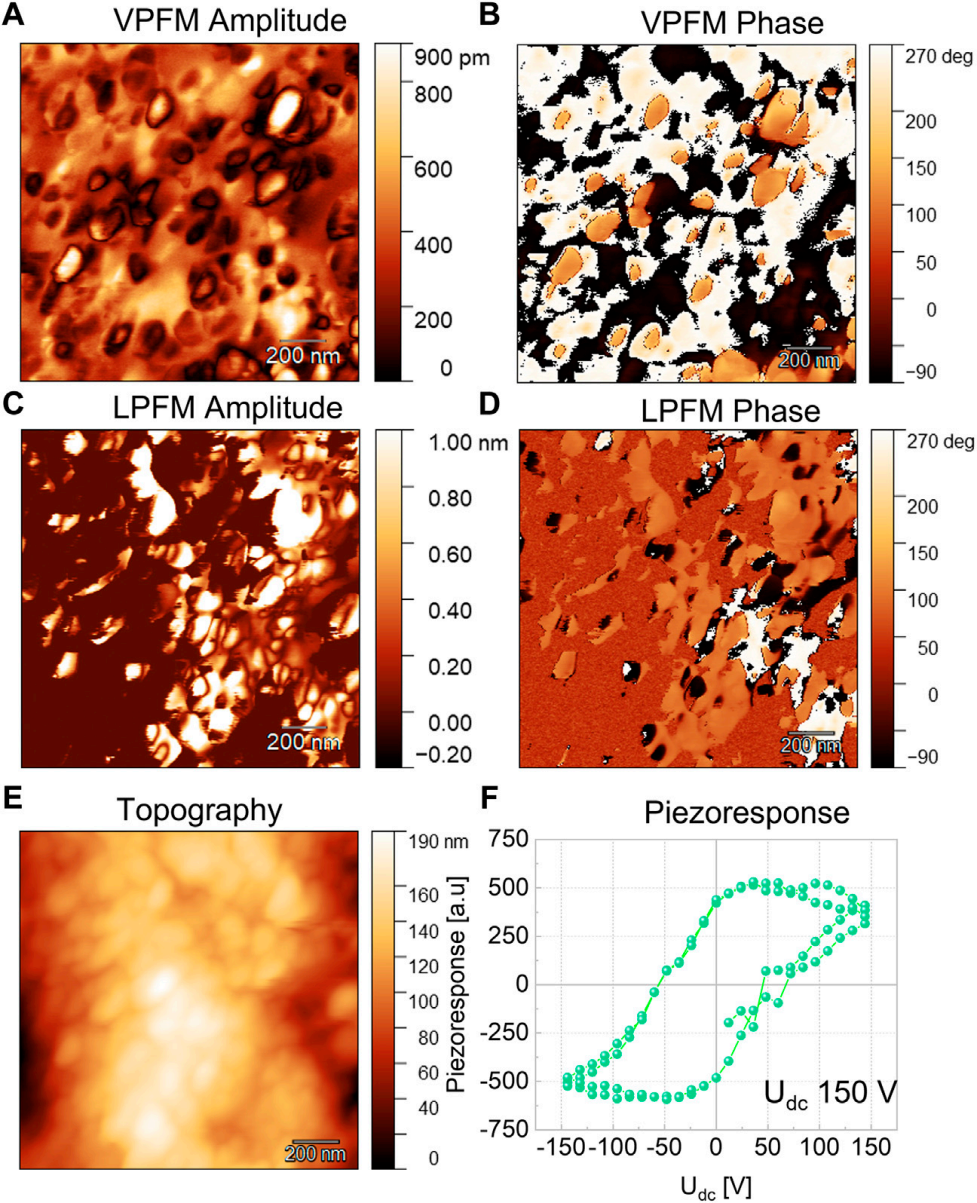
Representative PFM results for Nd and Mn co-doped BFO NPs. The vertical PFM amplitude **(A)**, and phase **(B)**, the lateral PFM amplitude **(C)**, and phase **(D)** images. The topography of NPs **(E)** and a local piezoresponse hysteresis loop **(F)**.

To measure ferroelectric and piezoelectric properties of the NPs, a dense sample is typically used, which is not easy to achieve when dealing with nanopowders while retaining their particle size. Compacted pellets usually have a low density and a large relative volume of voids and pores, which impedes application of sufficiently strong electric fields to the NPs and correct measurement of the generated strain or polarization. Hence, to probe the ferroelectricity in the NPs, we used piezoresponse force microscopy (PFM). PFM is a reliable method for studying ferroelectric and piezoelectric properties at the nanoscale. (Kalinin et al., 2007) (Castillo et al., 2013) The representative PFM results of Nd doped BFM NPs are shown in Figure 4 (PFM images of BFO, BFM NPs, Dy-BFM, Gd-BFM are reported elsewhere). (Dubey et al., 2020), Figure 4E shows the topography of the Nd-BFM NPs. Most of the NPs are agglomerated. Figures 4A,B show the corresponding vertical PFM (VPFM) amplitude and phase images, respectively. The VPFM signal stems from to the longitudinal piezoelectric effect and shows the distribution of the out of plane polarization component. Whereas the in-plane polarization component is shown by the lateral PFM (LPFM) amplitude and phase in Figures 4C,D. From the topography and phase images, most of the particles are found to be in a single-domain state. In the amplitude images the brighter zones correspond to the NPs with larger piezoresponse. (Shvartsman and Kholkin., 2011; Castillo et al., 2013). A local piezoresponse hysteresis loop for Nd-BFM at an applied DC voltage of 150 V is shown in Figure 4F. The change of the sign of the piezoresponse confirms that the polarization direction in the co-doped NPs can be switched by an electric field proving their ferroelectric state.

**FIGURE 5:**
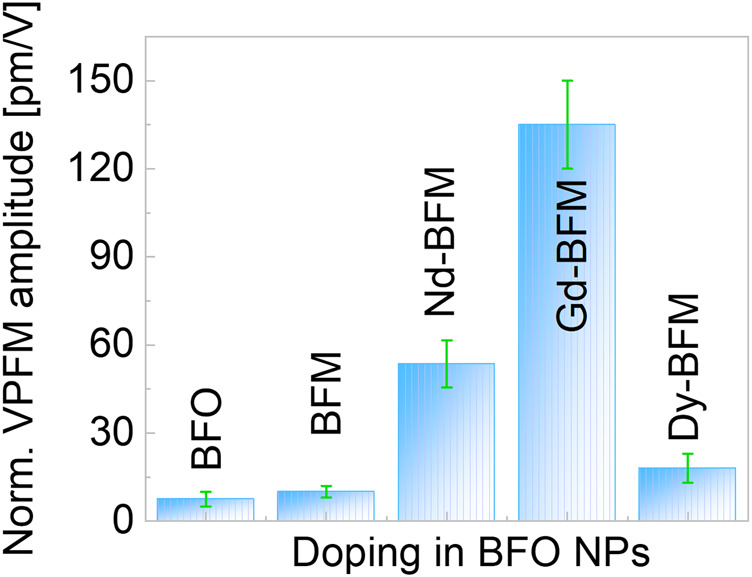
Normalised vertical PFM amplitude of undoped and doped BFO NPs.

For a comparison between the RE doped and the undoped BFM NPs, we normalized the VPFM piezoresponse to the probe voltage as shown in Figure 5. We have considered that the longitudinal piezoresponse depends on the crystallographic orientation of the particles with respect to the laboratory coordinate system. So, we expect that the NPs showing the maximum VPFM signal will have similar crystallographic orientation. We found that Gd-BFM has the highest piezoresponse, followed by Nd-BFM, whereas Dy-BFM has the smallest piezoresponse among the RE-doped samples studied. We observe that the doping with RE increases the piezoresponse of the BFM NPs. Since piezoresponse is proportional to the polarization value, it can be concluded that Bi-site doping in BFO NPs by RE elements increases the polarization in the following order: Gd^3+^ ˃ Nd^3+^ ˃ Dy^3+^. The alteration in polarization correlates with a doping induced alteration in crystal lattice parameters of pristine BFO NPs. It should be noted that the polarization of BFM NPs doped with Gd and Nd is larger than that of BFM NPs doped with Ag and alkaline earth metals (Dubey et al., 2019; Dubey et al., 2022). This indicates that moderated doping with rare earth elements improves the ferroelectric properties of BFO NPs, although the mechanism of this phenomena is unclear yet.

### Magnetic properties

**FIGURE 6:**
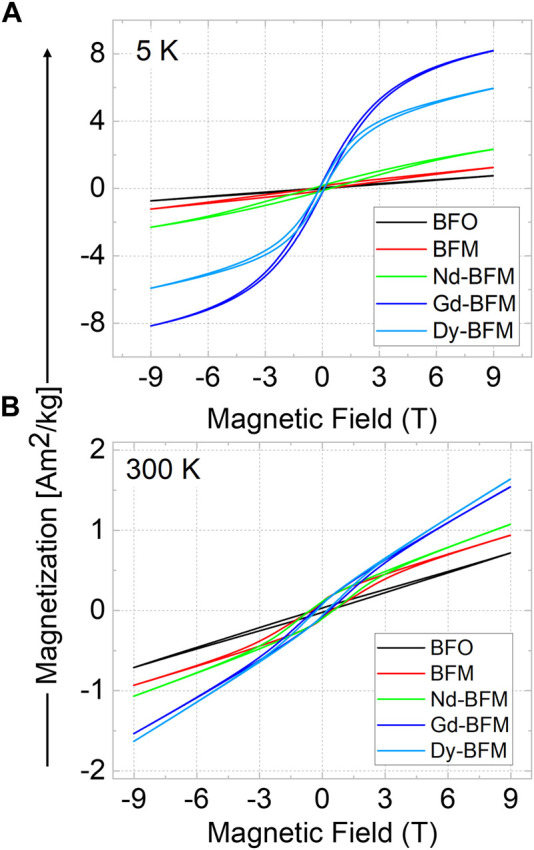
Magnetic hysteresis of pristine BFO, Mn-doped, and RE (Nd/Gd/Dy) and Mn co-doped BFO NPs at 5 K **(A)**, and at 300 K **(B)**.

Figures 6A,B show magnetization hysteresis loops*, M(H)*, measured at 5 K and at 300 K. The *M(H)* loops are not saturated up to the maximum applied field of 9 T. The maximum magnetization (*M*_max_), coercive field (*H*_C_), and remanent magnetization (*M*_r_) values at 300 K and at 5 K of the RE doped and undoped NPs are compared in Supplementary Table S3. As already known, 5% Mn doping widens the magnetic hysteresis and increases the *M*_r_ value. (Dubey et al., 2019), (Dubey et al., 2020) RE co-doping results in an additional increase of *M*_max_ and *M*_r_, which for Gd-BFM NPs reach 8.17 Am^2^/kg and 0.31 Am^2^/kg, at 5 K, respectively. After Gd, Dy is the second best RE dopant for the increase in magnetization of BFM NPs, whereas Nd is the last. Therefore, the order of increase in magnetization upon RE doping at the Bi site is BFO < BFM < Nd-BFM < Dy-BFM < Gd-BFM (at 5 K). The observed trend is in agreement with data reported in the literature. (Thakuria and Joy. 2010; Chang et al., 2017; Guo et al., 2010; Pattanayak et al., 2014) The shape of the hysteresis loops indicates a superposition of antiferromagnetic (AFM) and ferromagnetic (FM) contributions to the total magnetization, with the latter possibly being an effect of the dopants, in addition to the well-known weakly ferromagnetic property of small BFO nanoparticles.

For BFO and doped BFO NPs, *M(T)* measurements in the range of the Néel and Curie temperatures are rarely reported due to the decomposition of BFO at higher temperatures and the occurrence of secondary phases. Nevertheless, previously we have probed the phase stability of BFO NPs up to 1173 K using *in-situ* XRD and found out that BFO retains its pristine *R*3*c* crystal structure at least to 900 K under the XRD detection limit, (measurement time 62 min, NPs were maintained in an evacuated capsule at 10^−3^ mbar). Therefore, it was worth to probe temperature dependent magnetization in RE-doped BFO NPs up to 900 K at least. The temperature dependent magnetization was recorded for all samples at a magnetic field of 0.1 T from 300 K to 900 K (rising) and back down to 300 K (falling) as shown in Supplementary Figures S2, S3. For all samples, we observed that the magnetization rises above 630 K and reaches a maximum at about 750 K. Upon cooling, the magnetization continuously increases below 800 K. The magnetization values after cooling back to room temperature are approximately one order of magnitude greater than for as-synthesized NPs. The *M(H)* curves measured after this heating-cooling cycle (Supplementary Figure S3A) are qualitatively different from those measured before (Figure 6). The hysteresis loops have a pronounced S-shape with a remanent magnetization of 0.2–0.5 Am^2^/kg. Such behavior may indicate the formation of secondary magnetic phases due to partial decomposition of BFO above 630 K. Minor amounts of these phases lie below the XRD detection limit, but their ferrimagnetic response dominates the weak magnetic moment of BFO NPs. However, analysis of the measurements done upon heating below 630 K, gives information about the Néel temperature. For BFM NPs we observed an anomaly at the d*M*/d*T*(*T*) dependences around 575 K (Supplementary Figure S3C), which can be attributed to the antiferromagnetic-paramagnetic phase transition (Dubey et al., 2020). For the Dy-doped BMF NPs this anomaly is also around 575 K, but for Gd- and Nd-doped BMF NPs it shifts to 590 and 600 K, respectively. This indicates that these RE dopants stabilize the antiferromagnetic phase. It should be noted that the observed trend of the Néel temperature relative to the rare earth dopant correlates with the data reported in the literature (Thakuria and Joy. 2010), although the absolute values of the transition temperature are different. A more detailed discussion of temperature dependent magnetization curves is given in the supplementary information.

### Hyperthermia tests

**FIGURE 7:**
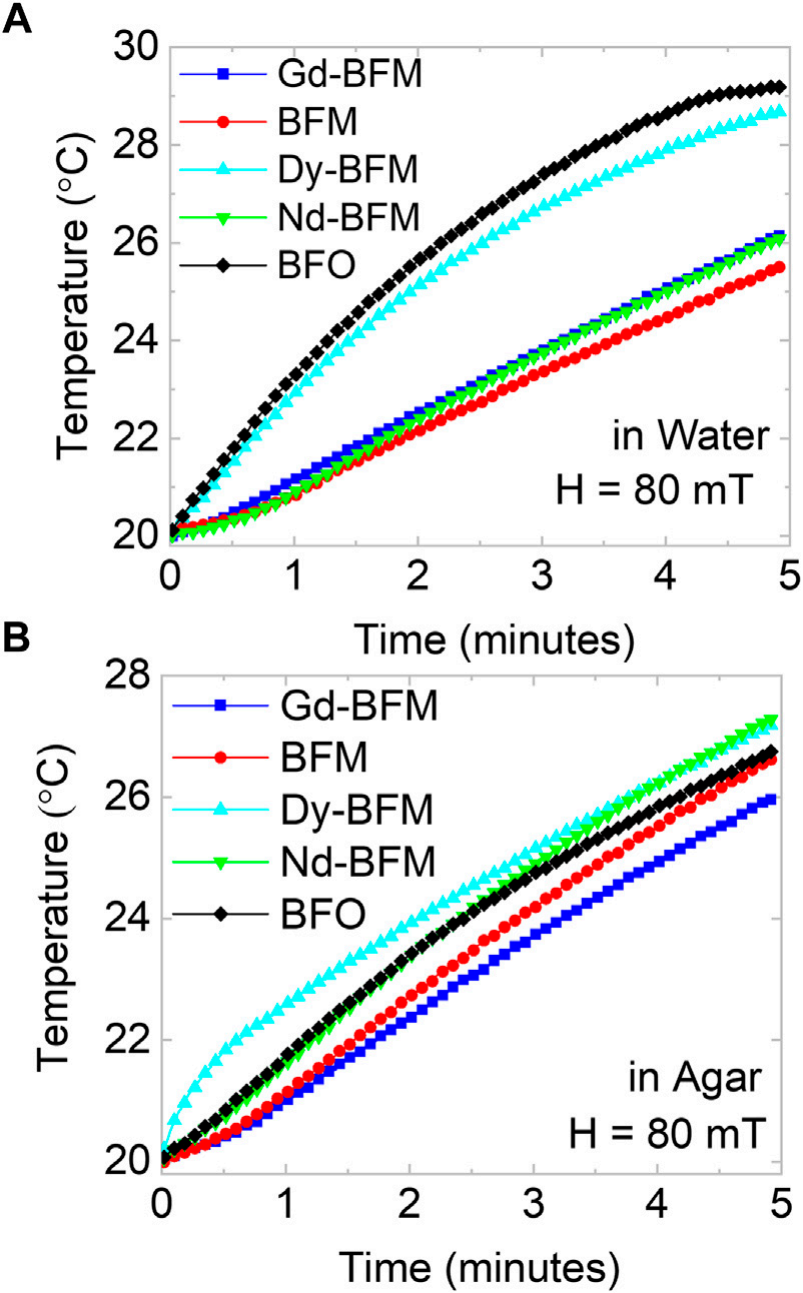
Hyperthermia tests of BFO and RE-Mn co-doped BFO NPs, the change in temperature due to applied AC field of 80 mT, in water **(A)**, and in agar **(B)** mediums.

For hyperthermia measurements, nanopowders were dispersed into water and agar medium by simply mixing at room temperature without any chemical or heat treatment. Figures 7A,B represent the change in temperature under an AC field of 80 mT field for samples with 1 mg/ml NPs concentration in both water and agar media. The NPs in water show the interaction of the system in a general medium and NPs in agar with a 2% weight solution enable the simulation of the activity of the NPs in blood, mimicking the environment inside the cells. Upon increasing the concentration from 1 mg/ml to 3 mg/ml, temperature rises faster in same time interval (5 min) as shown in Tables 1, 2.

**TABLE 1:** Maximum measured temperature produced by 3 mg/ml of doped and undoped BFO NPs dispersed into water medium at different applied AC magnetic fields.

NPs in water	Field (mT)	Maximum temperature (°C)
BFO	60	31.7
	80	38
BFM	60	31
	80	33.3
Gd-BFM	60	30.5
	80	37.5
Dy-BFM	60	30.2
	80	**39.6**
Nd-BFM	60	30.5
	80	36.2

Meaning of bold value shows reaching close to hyperthermia temperature window.

**TABLE 2:** Maximum measured temperature produced by 3 mg/ml of doped and undoped BFO NPs dispersed into Agar medium at different applied AC magnetic fields.

NPs in agar	Field (mT)	Maximum temperature (°C)
BFO	60	30.7
	80	37.2
BFM	60	32.3
	80	39
Gd-BFM	60	32
	80	38.6
Dy-BFM	60	31.3
	80	39.7
Nd-BFM	60	32
	80	38

3 mg/ml dispersion of NPs into water and agar mediums are studied and tabulated in Table 1 and Table 2, respectively at 60 and 80 mT. We observe that Dy-BFM could produce heat up to ∼39°C in water medium within 5 min. Similarly, 3 mg/ml dispersion of NPs in agar, manifests that a higher concentration of these magnetic NPs could heat more effectively than 1 mg/ml.

**FIGURE 8:**
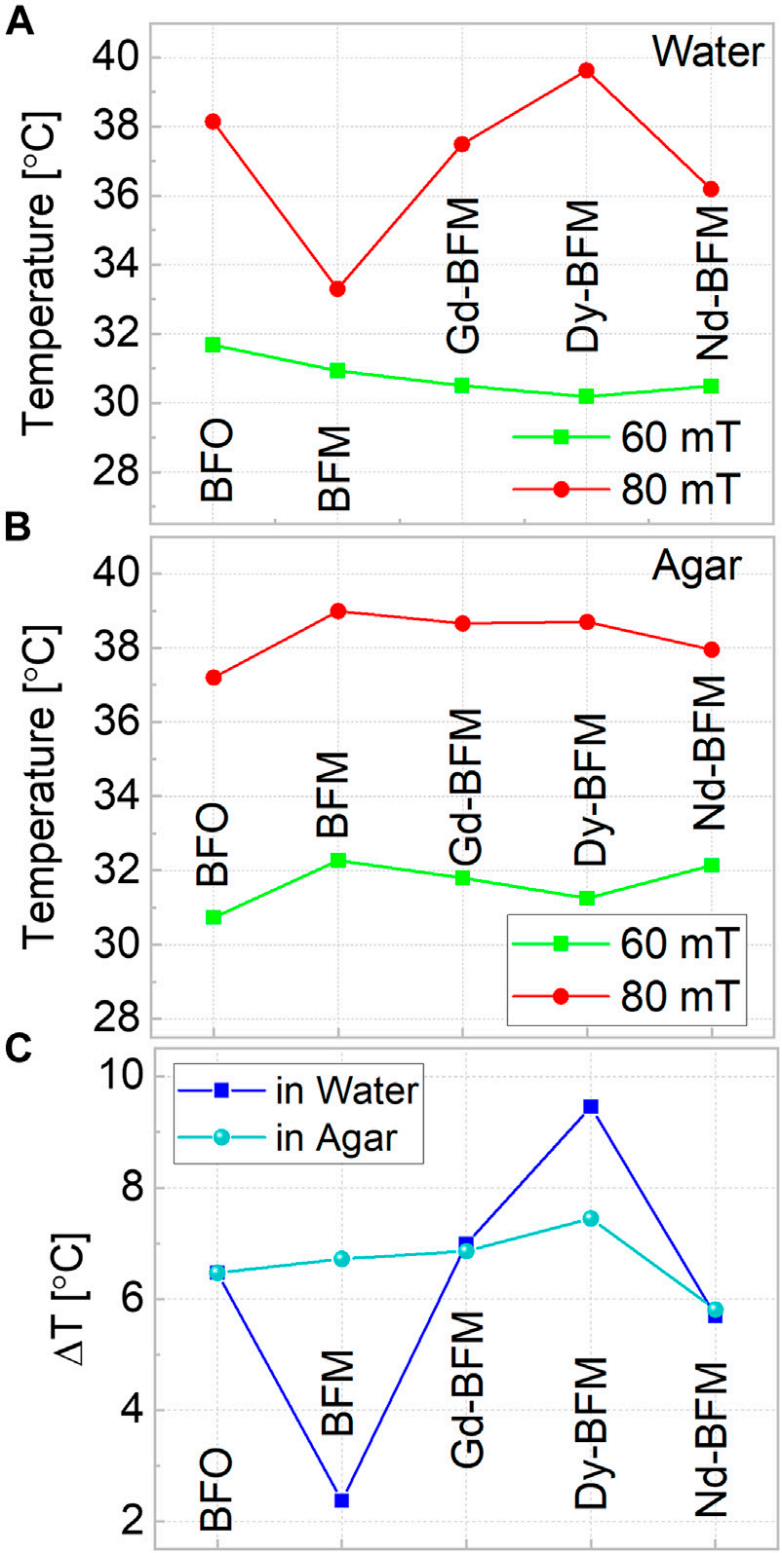
Hyperthermia tests at two different fields 60 mT and 80 mT of all doped and undoped BFO NPs, in water **(A)**, and in agar **(B)** and **(C)** shows the comparison of temperature difference in both mediums at 3 mg/ml concentrations.

In Figures 8A,B, the temperatures reached in 5 min by applying different AC fields are compared for all NPs in water and agar medium respectively. The difference in temperature raises by changing the fields for each type of NPs is plotted in Figure 8C. Doping of Gd, Dy, and Nd increases *ΔT* values as comparative to BFM NPs. In water medium upon increasing magnetic field, Dy-BFM NPs show more raise in temperature than Gd and Nd ones. The difference in heating efficiencies of RE-doped NPs might be due to their different shapes and saturation magnetization values. Dy-BFM NPs have higher *M*_max_ value at 300 K than other NPs, and their shape is irregular pentagonal (Dubey et al., 2022), which might be one of the factors behind its heating efficiency.

In agar, again Dy-BFM has best results. Among RE-doped samples, Nd-BFM produces the lowest temperature rise compared to other samples.

Though the therapeutic window was not reached in both the mediums, the Dy-BFM sample shows promising features with the potential to be further optimized in terms of chemical structure. The mentioned improvements in heating efficiencies can be evaluated using the equation for the Specific Absorption Rate (SAR) (Das et al., 2016);

$$SAR=\frac{\Delta T}{\Delta t}\frac{C_{P}}{\varphi }$$ (2)

where the Δ*T* gives the temperature change, Δ*t* gives the change in time, *C*_*P*_ denotes the specific heat capacity, and φ the mass of magnetic material per unit mass of liquid. We found that the SAR value is currently below 30 W/g for the Dy-BFM sample, which is highest among discussed NPs. SAR value depends upon the size, saturation magnetization, and magnetic anisotropy of NPs. Magnetic anisotropy can be tuned by changing shape of NPs or modifying the surface of NPs. Magnetic NPs with high SAR values are in demand for hyperthermia application purposes since they require lower amounts of material in order to reach the therapeutic temperature.. For spherical and cubic magnetite (Fe_3_O_4_) NPs 140 and 314 W/g SAR values have been reported, respectively. (Martinez-Boubeta et al., 2013) This shows that for doped-BFO NPs, different shapes of NPs must be studied in order to probe the effect of morphology on the SAR value.

Hyperthermia efficiency depends upon both magnetic and microstructural properties of NPs. RE-doped BFO NPs exhibit higher M_max_ values than BFM and BFO NPs at RT, which correlates with their improved hyperthermia performance Also, the morphology of RE-doped NPs differs from that of pristine BFO and BFM NPs. The peculiarity may lead to their different heating capabilities. In addition, the size of BFO/BFM NPs is reduced down to 22 nm due to RE doping, which can be a further factor for differences in heat production. The used concentrations of NPs in this research work are less compared to known magnetic NPs for hyperthermia, so concentration/amount of NPs can be increased in future studies. One can probe the effect of concentration of Dy-BFM NPs on the raise of temperature in water and agar mediums.

## Conclusion

Rare-earth (RE) elements (Nd, Gd, and Dy) are successfully substituted at Bi-sites, in BiFe_0.95_Mn_0.05_O_3_ NPs (BFM). The ligand free NPs are synthesized by a modified sol-gel route and are dispersed in water and agar media for testing the magnetic hyperthermia efficiency. The particle size, and morphology of NPs change as per different RE dopants. We found elliptical morphology for Nd-BFM NPs, rectangular for Gd-BFM, and irregular hexagonal morphology for Dy-BFM, respectively. The RE doped NPs consist of a single ferroelectric domain and show larger piezoresponse than both pure BFO and Mn-doped BFO NPs. We found that Gd doping is best for improvement in piezoresponse and corresponding ferroelectricity in BFM NPs. Magnetometry results show an increase of the maximum magnetization of BiFe_0.95_Mn_0.05_O_3_ NPs (BFM) up to 550% at 300 K *via* introducing Gd. The observed trend in the magnetic properties (magnetization and Néel temperature) with respect to the rare earth dopant correlates with the data presented in the literature. Only 1 mg/ml dispersion of NPs into water and agar can increase the temperature of dispersion under applied AC magnetic field of 60–80 mT. We observed that Dy-BFM dispersion in water, can heat up to ∼39°C at 80 mT, which is close to the hyperthermia window of 40–45°C required for hyperthermia tests as per the standards. These results indicate that an increased concentration of NPs can make them suitable for hyperthermia tests and other biomedical applications.

## Data Availability

The original contributions presented in the study are included in the article/Supplementary Material, further inquiries can be directed to the corresponding author.
