# Supplementary material for: Rare-earth doped BiFe0.95Mn0.05O3 nanoparticles for potential hyperthermia applications
Source: Front Bioeng Biotechnol. 2022 Oct 18;10:965146. doi: 10.3389/fbioe.2022.965146 (PMC9623096; doi:10.3389/fbioe.2022.965146)
Supplement: Supplementary file 1 [file DataSheet1.docx]

Supplementary Material:

Rare-Earth Doped BiFe_0.95_Mn_0.05_O_3_ Nanoparticles for Potential Hyperthermia Applications

Astita Dubey,^1^ Soma Salamon,^2^ Supun B. Attanayake,^3^ Syaidah Ibrahim,^1^ Joachim Landers,^2^ Marianela Escobar Castillo,^1^ Heiko Wende,^2^ Hari Srikanth,^3^ Vladimir V. Shvartsman,^1^ Doru C. Lupascu^1^

^1^Institute for Materials Science and Center for Nanointegration Duisburg-Essen (CENIDE), University of Duisburg-Essen, 45141 Essen, Germany

^2^Faculty of Physics and Center for Nanointegration Duisburg-Essen (CENIDE), University of Duisburg-Essen, 47057, Duisburg, Germany

^3^Department of Physics, University of South Florida, Tampa, FL 33620, USA

*** Correspondence:**Astita Dubey
[astita.dubey@uni-due.de](mailto:astita.dubey@uni-due.de)

# *EDXS analysis:*

Table S 1: Elemental analysis via atomic percent in doped BFO NPs.

| **Elements (At%)** | **BFO**^1^ | **BFM**^1^ | **Nd-BFM** | **Gd-BFM**^2^ | **Dy-BFM**^3^ |
| --- | --- | --- | --- | --- | --- |
| Bi | 15.4 | 21.2 | 16.1 | 8.7 | 11.47 |
| Fe | 15.7 | 21.1 | 16.2 | 8.5 | 11.62 |
| Mn | - | 1.2 | 1.0 | 0.4 | 0.59 |
| Nd/Gd/Dy | - | - | 1.0 | 0.4 | 0.51 |
| O | 68.8 | 56.5 | 65.7 | 36.4 | 75.5 |

# *Particle size analysis:*

Table S 2: Particle size comparison of pristine and doped BFO NPs.

|  | **BFO**^1^ | **BFM**^1^ | **Nd-BFM** | **Gd-BFM**^2^ | **Dy-BFM**^3^ |
| --- | --- | --- | --- | --- | --- |
| *Size [nm]* | 46 ± 3 | 35 ± 6 | 22 ± 3 | 28 ± 4 | 22 ± 4 |
| *Morphology* | cuboidal | spherical | elliptical | Irregular-rectangular | Irregular-pentagon |

# *Magnetic data analysis:*

Table S 3: Comparison of magnetic values of doped and undoped BFO NPs at 5 K and 300 K.

| Sample | *M_max_* (Am^2^/kg) | | *M_r_* (Am^2^/kg) | | *μ*_0_*H_C_* (T) | |
| --- | --- | --- | --- | --- | --- | --- |
|  | **300 K** | **5 K** | **300 K** | **5 K** | **300 K** | **5 K** |
| BFO | 0.713 | 0.715 | 0.030 | 0.031 | 0.313 | 0.300 |
| 5 Mn | 0.932 | 1.234 | 0.097 | 0.126 | 0.473 | 0.471 |
| 5 Nd 5 Mn | 1.060 | 2.310 | 0.110 | 0.158 | 0.552 | 0.447 |
| 5 Gd 5 Mn | 1.540 | 8.170 | 0.080 | 0.305 | 0.321 | 0.129 |
| 5 Dy 5 Mn | 1.634 | 5.930 | 0.068 | 0.246 | 0.248 | 0.115 |

***Discussion on temperature dependent magnetic curves of BFO and doped BFO NPs:***

The comparison between the rising and falling *M(T)* curves for pure and doped BFO clearly shows an irreversible behavior.

For the rising (300-900 K) *M(T)* curve of the BFO sample [Figure S2], a broad peak is observable between 620 and 820 K, pointing towards the formation of a phase with high net magnetization. In the same temperature region, the falling curve displays lower magnetization, which could indicate that the forming phase is unstable at the higher temperatures that the sample was subjected to. The descending (900-300K) curve shows clear Brillouin behavior, with the magnetization beginning to rise below 820 K (*T*_C1_), further rising monotonically towards 300 K. This could be explained by the formation of a minute amount of ferrimagnetic Fe-oxide such as magnetite (*T*_C_ = 850 K) or maghemite (*T*_C_ = 820-986 K).^4^ Considering the magnetization of < 0.5 Am^2^/kg at 300 K and 0.1 T, this value could be due to less than 1 wt % of the total sample material, making it difficult to be discerned in XRD data. A close examination of the curve indicates a further anomaly located at near 610-620 K, with a minuscule contribution to the magnetization, which may correspond to the Néel temperature of pure BFO nanoparticles and this transition has also been observed before in the calorimetry measurements.^1^

Similar for the BFM NPs, the Néel temperature is near 575 K, and this shifts to a higher temperature for the Gd and Nd doped BFM NPs. However, for Dy-BFM NPs the Néel temperature value remains similar to the BFM NPs.


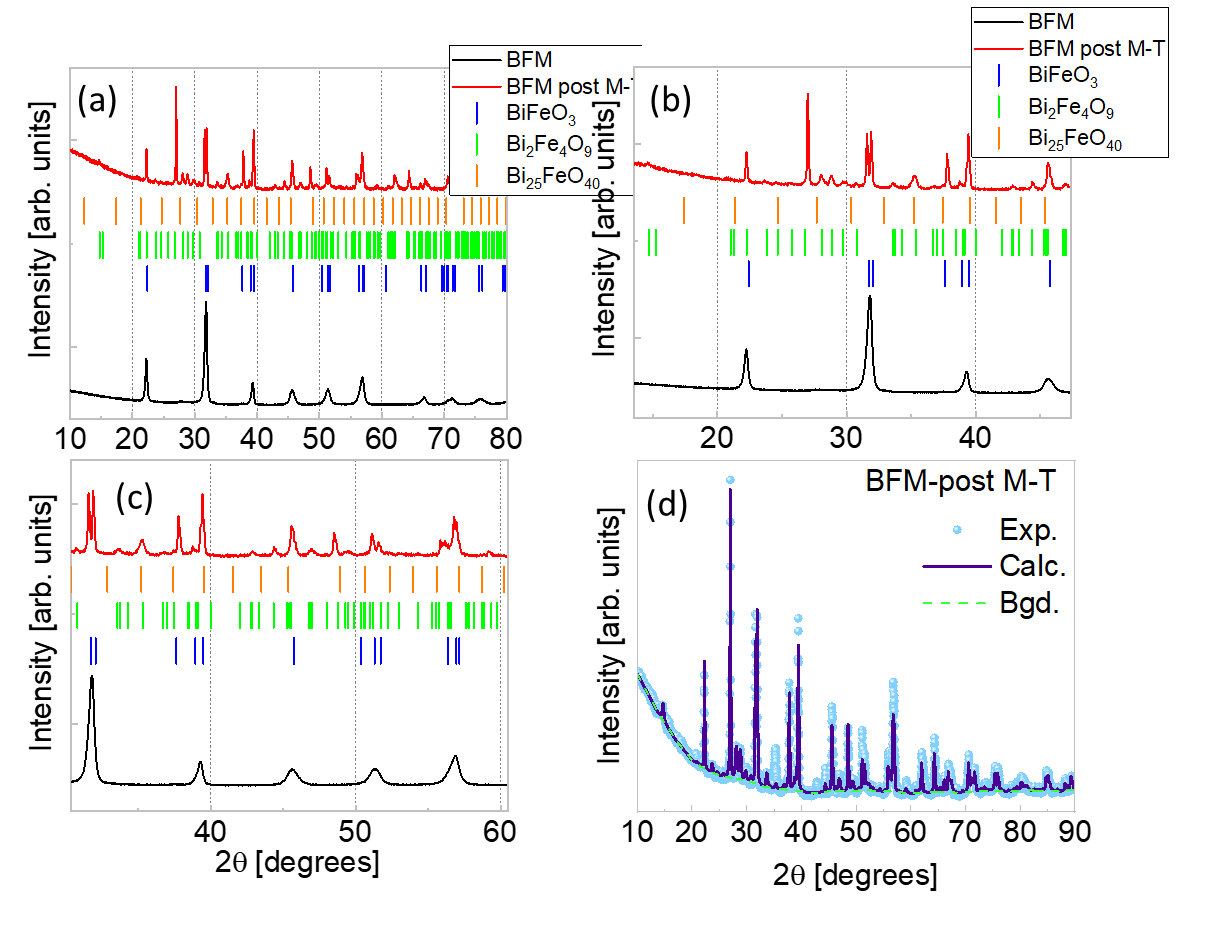
Figure S**1**: X-ray diffraction analysis after temperature dependent magnetic measurements of 5 mol % Mn doped BFO NPs. Comparison of XRD before and after *M***(***T***) measurements** along with three phases (a), magnified graphs in 10-45 and 35-60 2θ range (b, c). The Rietveld refinement fit (d).

To check the phase content of the NPs after heating up to 1000 K we have measured XRD diffractograms. In Figure S1, the representative XRD phase analysis of BFM NPs is shown, where the XRD diffractograms are collected after *M(T)* measurements. For the rest of the samples XRD patterns post M-T are shown in Figure S4. As per the phase analysis we could fit the experimental peaks of the BFM sample by three different phases including *R*3*c*: BiFe_0.05_Mn_0.05_O_3_, *I*23: Bi_25_FeO_39_ (sillenite), *Pbam*: Bi_2_Fe_4_O_9_ (mullite) as shown in Figures S1b and S1c. There is a good match found between the experimental pattern and calculated fit pattern done using Rietveld refinement with a *R*_wp_ value 6 and goodness of fit 1.3. [Figure S1d]

The decomposition of BFO has been reported previously as follows.^5^

|  | ${49BiFeO}_{3}\to{Bi}_{25}{FeO}_{39}+{12Bi}_{2}{{Fe}_{4}O}_{9}$ | (1) |
| --- | --- | --- |

Both sillenite and mullite exhibit relatively limited saturation magnetization similar to BFO NPs.^6^, ^7^, ^8^ In literature, for mullite and sillenite magnetic ordering is reported below *T*_N_ = 260 K ^9^ and 272 K, ^10^ respectively. Thereby, they are paramagnetic in the temperature region studied here and do not contribute considerably to the characteristic features described above.


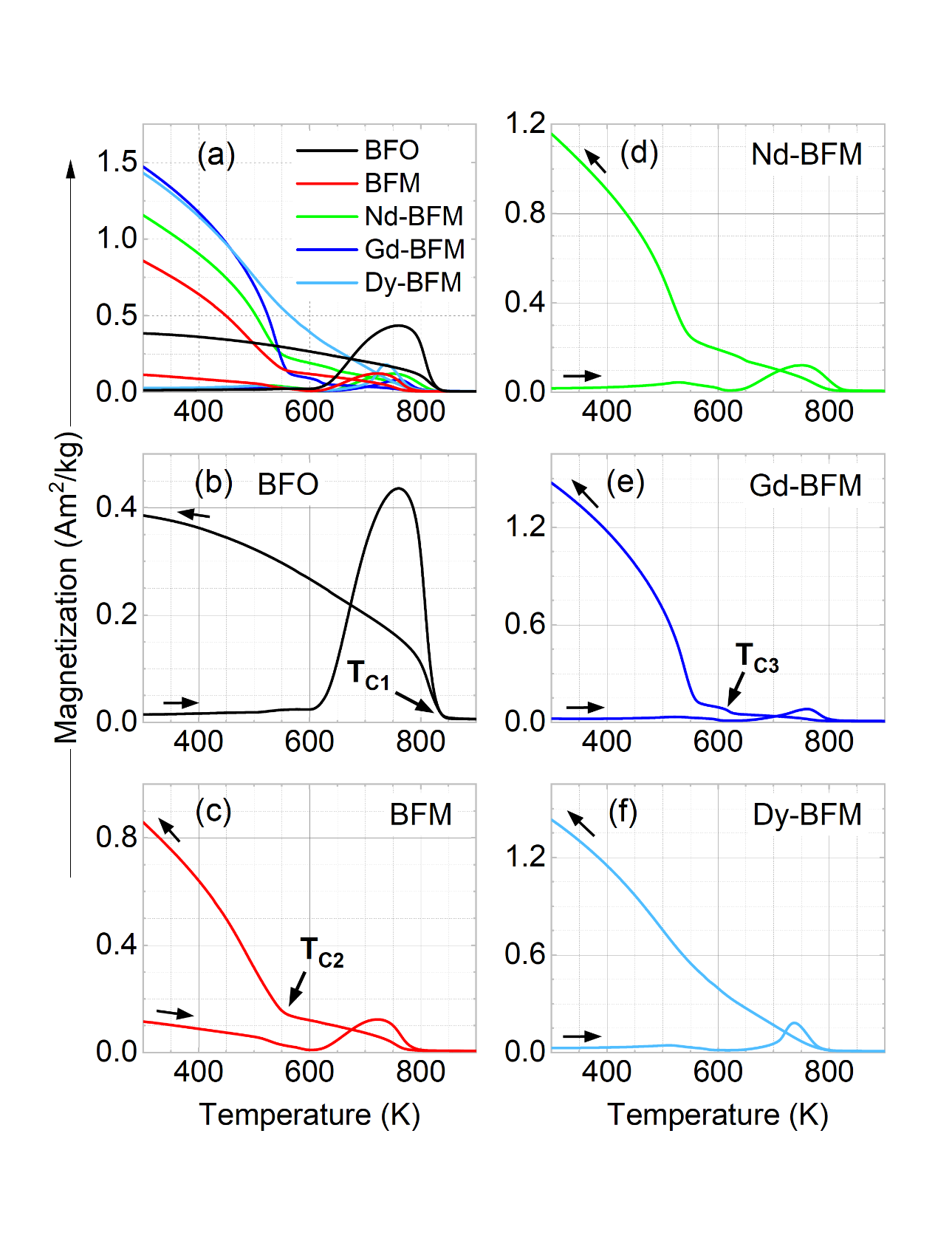


Figure S **2**: Temperature dependent magnetization plots for all doped and undoped BFO NPs up to 900 K, at 0.1 T. Comparison of all NPs (a), individual *M*(*T*) curves with characteristic features being highlighted (b-f).

We can thus conclude that a minute Fe-oxide phase has formed with a small contribution to the overall magnetization, which could explain the quickly saturating *M(H)* curve of the heated pure BFO sample compared to the pre-heating state [Figure S3a]. In general, this behaviour is consistent among the Mn and RE-doped BFM samples, albeit with a slightly reduced *T*_C1_, presumably caused by the addition of Mn to the ferrimagnetic Fe-oxide byphase. ^11^

When comparing the BFO with the BFM sample [Figure S3], a further additional magnetic phase transition can be seen. The aforementioned Fe-oxide phase is still present, and it is visible just below 800 K (*T*_C1_), however, a second phase is also present, with a lower transition temperature of 540 K (*T*_C2_).


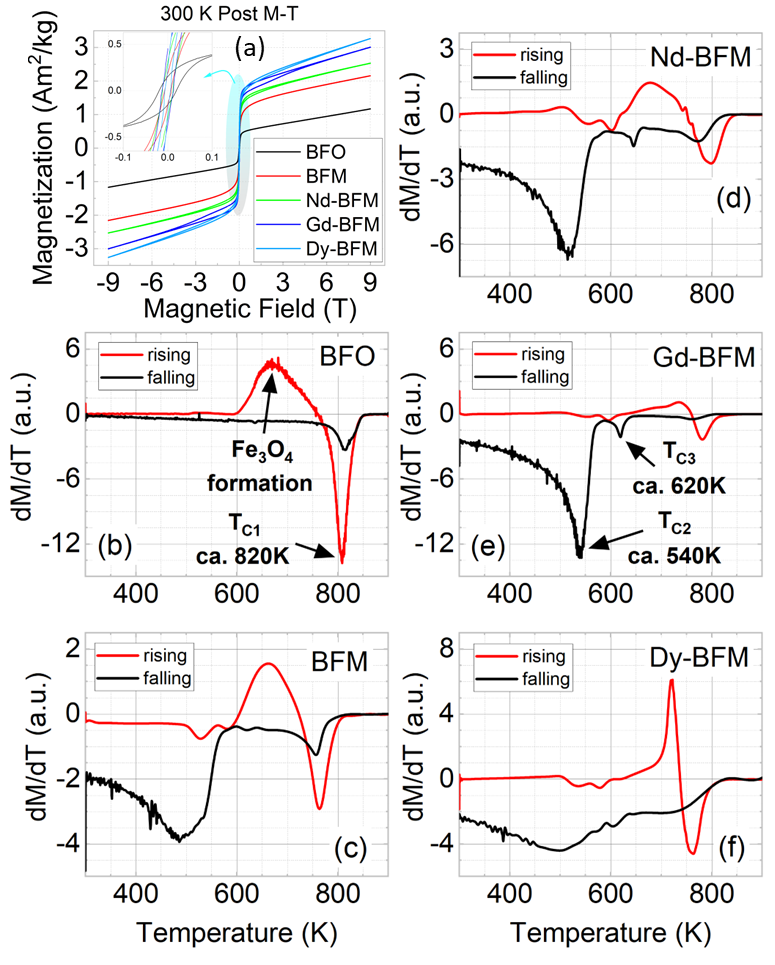


Figure S3: Magnetic hysteresis of pristine BFO, Mn-doped, and RE (Nd/Gd/Dy) and Mn co-doped BFO NPs at 300 K, after high temperature *M*(*T*) measurements (a). Derivative of magnetization curves with respect to temperature (dM/dT) for individual samples with marked transition temperatures (b-f) for rising (red) and falling *M*(*T*) sweep (black). For better readability, derivatives have been subjected to a smoothing function, while making sure to fully preserve the highlighted characteristic features.

**
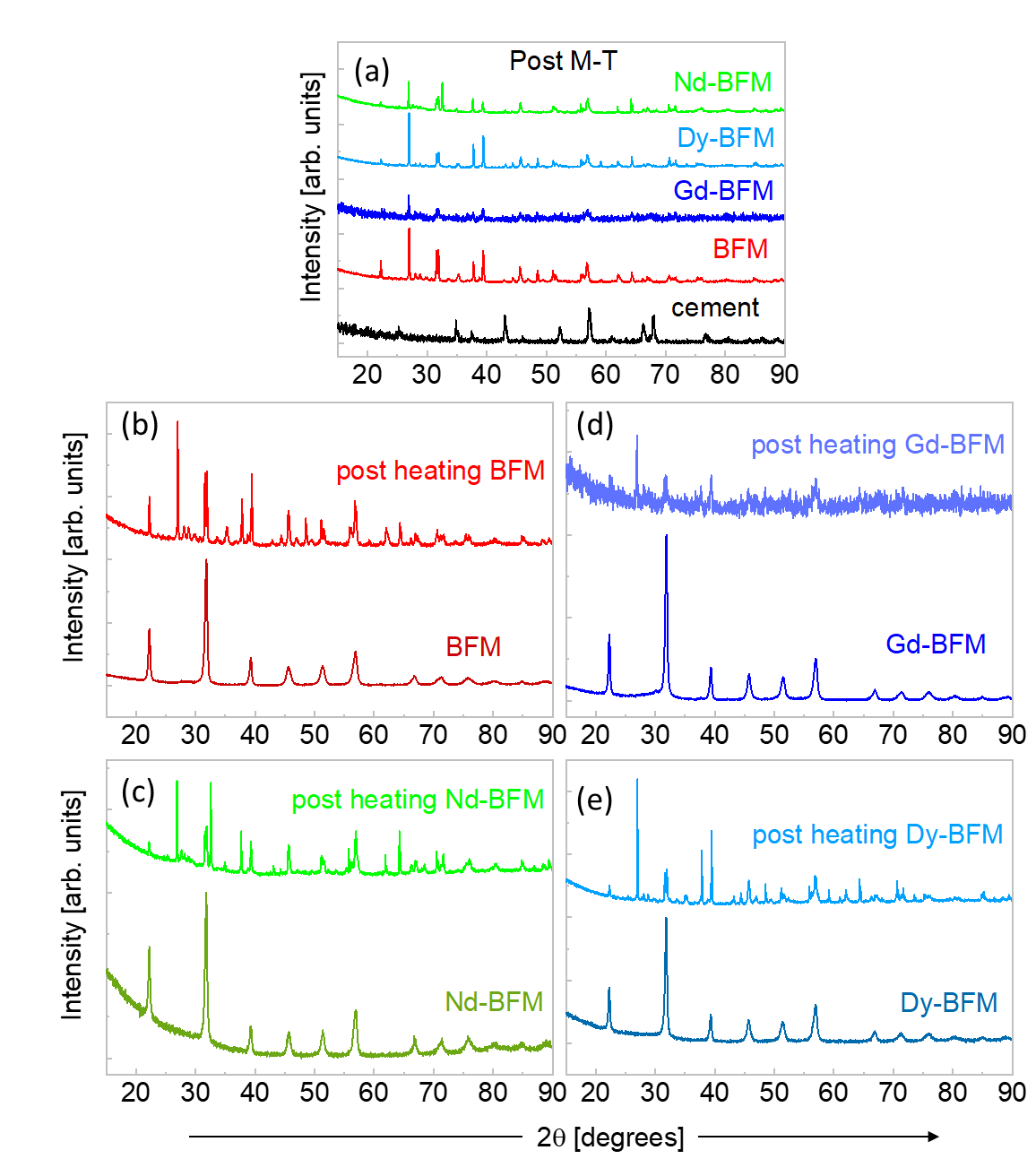
**

Figure S4: XRD diffractograms of doped BFO NPs after temperature dependent magnetic measurements. The comparison of patterns with used cement while measurements (a), XRD before and after *M(T)* measurements for Mn-doped BFO NPs (b), of Nd-BFM (c), Gd-BFM (d), and for Dy-BFM NPs (e).

The results show that the formation of magnetic phases during temperature dependent magnetic measurements are challenging to deal with in order to study the magnetic behavior of pristine BFO or BFM NPs at high temperatures. However, we could estimate the Néel temperature of BFM and RE-doped BFM NPs using derivative of *M*-*T* curves.

**References:**

(1) Dubey, A.; Escobar Castillo, M.; Landers, J.; Salamon, S.; Wende, H.; Hagemann, U.; Gemeiner, P.; Dkhil, B.; Shvartsman, V. V.; Lupascu, D. C. Effect of Mn and Ba Codoping on a Magnetic Spin Cycloid of Multiferroic Bismuth Ferrite Nanoparticles. *J. Phys. Chem. C* **2020**, *124* (40), 22266–22277. https://doi.org/10.1021/acs.jpcc.0c05778.

(2) Dubey, A.; Moltó, I. S.; Castillo, M. E.; Andronescu, C.; Lupascu, D. C. Electrochemical Activity of Doped BiFeO3 Nanoparticles towards Hydrogen Evolution Reaction (HER). *Submitted* **2022**.

(3) Dubey, A.; Keat, C. H.; Shvartsman, V. V.; Yusenko, K. V.; Escobar, M. C.; Hagemann, A. G. B. U.; Kovalenko, S. A.; Stähler, J.; Lupascu, D. C. Mono-, Di-, and Tri-Valent Cation Doped BiFe0.95Mn0.05O3 Nanoparticles: Ferroelectric Photocatalysts. *submitted* **2022**.

(4) Schwertmann, U.; Cornell, R. M. *Wiley VCH*; Wiley, 2003. https://doi.org/10.1002/3527602097.

(5) Carvalho, T. T.; Tavares, P. B. Synthesis and Thermodynamic Stability of Multiferroic BiFeO3. *Mater. Lett.* **2008**, *62*, 3984–3986. https://doi.org/10.1016/j.matlet.2008.05.051.

(6) Bernardo, M. S. Synthesis, Microstructure and Properties of BiFeO3-Based Multiferroic Materials: A Review. *Bol. la Soc. Esp. Ceram. y Vidr.* **2014**, *53* (1), 1–14. https://doi.org/10.3989/cyv.12014.

(7) Salak, A. N.; Cardoso, J. P. V.; Vieira, J. M.; Shvartsman, V. V.; Khalyavin, D. D.; Fertman, E. L.; Fedorchenko, A. V.; Pushkarev, A. V.; Radyush, Y. V.; Olekhnovich, N. M.; Tarasenko, R.; Feher, A.; Čižmár, E. Magnetic Behaviour of Perovskite Compositions Derived from BiFeO3. *Magnetochemistry* **2021**, *7* (11), 1–12. https://doi.org/10.3390/magnetochemistry7110151.

(8) Palai, R.; Katiyar, R. S.; Schmid, H.; Tissot, P.; Clark, S. J.; Robertson, J.; Redfern, S. A. T.; Catalan, G.; Scott, J. F. Alpha Phase and Gamma-Beta Metal-Insulator Transition in Multiferroic BiFeO3. *Phys. Rev. B* **2008**, *77* (1), 014110. https://doi.org/10.1103/PhysRevB.77.014110.

(9) Singh, A. K.; Kaushik, S. D.; Kumar, B.; Mishra, P. K.; Venimadhav, A.; Siruguri, V.; Patnaik, S. Substantial Magnetoelectric Coupling near Room Temperature in Bi2Fe4O9. *Appl. Phys. Lett.* **2008**, *92* (13), 2–5. https://doi.org/10.1063/1.2905815.

(10) Jebari, H.; Tahiri, N.; Boujnah, M.; El Bounagui, O.; Taibi, M.; Ez-Zahraouy, H. Theoretical Investigation of Electronic, Magnetic and Magnetocaloric Properties of Bi25FeO40 Compound. *Phase Transitions* **2021**, *94* (3–4), 147–158. https://doi.org/10.1080/01411594.2021.1931690.

(11) Amighian, J.; Karimzadeh, E.; Mozaffari, M. The Effect of Mn2+ Substitution on Magnetic Properties of MnxFe3-XO4 Nanoparticles Prepared by Coprecipitation Method. *J. Magn. Magn. Mater.* **2013**, *332*, 157–162. https://doi.org/10.1016/j.jmmm.2012.12.005.

(12) Dhanalakshmi, B.; Pratap, K.; Parvatheeswara Rao, B.; Rao, P. S. V. S. Effects of Mn Doping on Structural, Dielectric and Multiferroic Properties of BiFeO3 Nanoceramics. *J. Alloys Compd.* **2016**, *676*, 193–201. https://doi.org/10.1016/j.jallcom.2016.03.208.

(13) Varshney, D.; Kumar, A.; Verma, K. Effect of A Site and B Site Doping on Structural, Thermal, and Dielectric Properties of BiFeO3 Ceramics. *J. Alloys Compd.* **2011**, *509* (33), 8421–8426. https://doi.org/10.1016/j.jallcom.2011.05.106.
